# ASM Journals Eliminate Impact Factor Information from Journal Websites

**DOI:** 10.1128/mSphere.00184-16

**Published:** 2016-07-11

**Authors:** Arturo Casadevall, Stefano Bertuzzi, Michael J. Buchmeier, Roger J. Davis, Harold Drake, Ferric C. Fang, Jack Gilbert, Barbara M. Goldman, Michael J. Imperiale, Philip Matsumura, Alexander J. McAdam, Marcela F. Pasetti, Rozanne M. Sandri-Goldin, Thomas Silhavy, Louis Rice, Jo-Anne H. Young, Thomas Shenk

**Affiliations:** a Department of Molecular Microbiology and Immunology, Johns Hopkins Bloomberg School of Public Health, Baltimore, Maryland, USA; b American Society for Microbiology (ASM), Washington, DC, USA; c Department of Molecular Biology and Biochemistry, University of California, Irvine, Irvine, California, USA; d Howard Hughes Medical Institute, Chevy Chase, Maryland, USA, and Program in Molecular Medicine, University of Massachusetts Medical School, Worcester, Massachusetts, USA; e Department of Ecological Microbiology, University of Bayreuth, Bayreuth, Germany; f University of Washington School of Medicine, Seattle, Washington, USA; g Department of Surgery, University of Chicago, Chicago, Illinois, USA; h Department of Microbiology and Immunology, University of Michigan, Ann Arbor, Michigan, USA; i Department of Microbiology and Immunology, University of Illinois at Chicago, Chicago, Illinois, USA; j Boston Children’s Hospital and Harvard Medical School, Boston, Massachusetts, USA; k Center for Vaccine Development and Department of Pediatrics, University of Maryland, Baltimore, Maryland, USA; l Department of Microbiology and Molecular Genetics, University of California, Irvine, California, USA; m Department of Molecular Biology, Princeton University, Princeton, New Jersey, USA; n Departments of Medicine and Microbiology and Immunology, Warren Alpert Medical School of Brown University, Providence, Rhode Island, USA; o Department of Medicine, University of Minnesota, Minneapolis, Minnesota, USA

## Abstract

Many scientists attempt to publish their work in a journal with the highest possible journal impact factor (IF). Despite widespread condemnation of the use of journal IFs to assess the significance of published work, these numbers continue to be widely misused in publication, hiring, funding, and promotion decisions (1, 2).

## EDITORIAL

Many scientists attempt to publish their work in a journal with the highest possible journal impact factor (IF). Despite widespread condemnation of the use of journal IFs to assess the significance of published work, these numbers continue to be widely misused in publication, hiring, funding, and promotion decisions (1, 2).

There are a number of problems with this approach. First of all, the journal IF is a journal-level metric, not an article-level metric, and its use to determine the impact of a single article is statistically flawed since citation distribution is skewed for all journals, with a very small number of articles driving the vast majority of citations (3, 4). Furthermore, impact does not equal importance (5) or advancement to the field, and the pursuit of a high IF, whether at the article or journal level, may misdirect research efforts away from more important priorities. The causes for the unhealthy obsession with IF are complex (2). High-IF journals limit the number of their publications to create an artificial scarcity and generate the perception that exclusivity is a marker of quality. The relentless pursuit of high-IF publications has been detrimental for science (2, 5). This behavior is an example of the economic phenomenon known as the “tragedy of the commons” (6), in which individuals engage in a behavior that benefits them individually at the expense of communal interests. Individual scientists receive disproportionate rewards for articles in high-IF journals, but science as a whole suffers from a distorted value system, delayed communication of results as authors shop for the journal with the highest IF that will publish their work, and perverse incentives for sloppy or dishonest work (2). Since many investigators consider IFs in deciding where to submit their manuscripts, many journals list their IFs on their websites, and until now American Society for Microbiology (ASM) journals have been no exception.

ASM journals focus on publishing high-quality science that has been rigorously peer reviewed by experts and evaluated by academic editors. The primary mission of ASM is to advance microbial science. At the recent Journals Board meeting that took place during ASM Microbe 2016 in Boston, MA, the editors in chief and the ASM leadership decided to no longer advertise the IFs of ASM journals (7).

Our goal is to avoid contributing further to the inappropriate focus on journal IFs. Although this action by itself may have little effect on a practice that is deeply entrenched in the biological sciences, we hope that removing IFs from ASM journal websites makes a statement of principle that will be emulated by other journals.
